# Chlorhexidine residues in sludge from municipal wastewater treatment plants: analytical determination and toxicity evaluation

**DOI:** 10.1007/s00216-022-04214-0

**Published:** 2022-07-13

**Authors:** Miguel Cobo Golpe, Gabriela Castro, Maria Ramil, Rafael Cela, Ysabel Santos, Isaac Rodríguez

**Affiliations:** 1 Department of Analytical Chemistry, Nutrition and Food Sciences, IAQBUS - Institute of Research On Chemical and Biological Analysis, Universidade de Santiago de Compostela, R/Constantino Candeira SN, 15782 Santiago de Compostela, Spain; 2 Department of Microbiology and Parasitology, IAQBUS- Institute of Research On Chemical and Biological Analysis, Universidade de Santiago de Compostela, R/Constantino Candeira SN, 15782 Santiago de Compostela, Spain

**Keywords:** Chlorhexidine, Matrix solid-phase extraction, Liquid chromatography tandem mass spectrometry, Sludge, Ecotoxicity

## Abstract

In this work, a procedure for the sensitive and selective determination of chlorhexidine in sludge from municipal sewage treatment plants (STPs) based on matrix solid-phase dispersion (MSPD) and liquid chromatography coupled to tandem mass spectrometry (LC–MS/MS) was optimized and validated. Analysis of sewage sludge samples, obtained from different STPs in Northwest Spain from 2018 to 2021, showed that chlorhexidine was ubiquitous in this environmental compartment with concentrations between 0.3 and 16 µg g^−1^. The toxicity of this pollutant was assessed in in vitro assays considering three different model organisms: *Candida albicans*, *Escherichia coli*, and *Staphylococcus aureus*. *C. albicans* was the most sensitive of the tested microorganisms to chlorhexidine with a lethal threshold concentration of 0.1 mg L^−1^. Thus, the lowest observed sludge residue was 3 times higher than the acute toxicity threshold measured for *C. albicans.* Moreover, *E. coli* and *S. aureus* were also affected at chlorhexidine concentrations around 1.8 mg L^−1^ and 0.5 mg L^−1^, respectively. So, chlorhexidine residues might affect the population of microorganisms existing in STPs. In addition, the potential phytotoxicity of the compound was evaluated with germination experiments using different model seeds. At the evaluated dose (10 µg g^−1^ dried soil), chlorhexidine did not affect the germination of *Sorghum saccharatum*, *Lepidium sativum*, or *Sinapis alba* seeds. Thus, amending agriculture soils with chlorhexidine containing sludge is unlikely to affect the germination of plants.

**Supplementary Information:**

The online version contains supplementary material available at 10.1007/s00216-022-04214-0.

## Introduction

Emerging contaminants have been a concerning issue over the last two decades [1, 2]. These compounds comprise from pharmaceuticals to personal care products or high production volume chemicals. All of them are discharged in sewage and are only partially eliminated in sewage treatment plants (STPs) [3, 4]. Thus, they are introduced in the environment and in the water cycle causing different harmful effects to the wildlife [5–7].

Antiseptics and disinfectants present an increasing consumption in the last years and they have become target micropollutants in the aquatic environment [8]. Among them, chlorhexidine is employed as biocide and disinfectant in personal care, particularly mouth washes [9, 10], and household products [11]. It has been proven to be toxic to some animals such as rats by accidental ingestion [12], and it has been recently revealed to interfere with human sex hormone receptor pathways [13]. Moreover, chlorhexidine has already been reported as toxic for some aquatic organisms such as *P. subcapitata*, *D. magna*, *D. rerio*, and *V. fischeri* [14]. Effluent water from STPs contains just 1–2% of the total mass of chlorhexidine entering treatment plants [15–17]. However, owing to its large molecular weight, limited water solubility, and poor degradability [18, 19], this compound is expected to be accumulated in the sludge fraction of STPs.

As it has been already reported for antibiotics [20], biocides might also contribute to the development of resistant microorganisms [21]. In the environment of STPs, chlorhexidine has proved to be toxic for the microbiota involved in the biodegradation of organic pollutants [21]. In this vein, Keerthisinghe et al. [21] have recently reported that chlorhexidine at environmentally relevant levels could lead to a significant reduction of *Comamonadaceae* and *Flavobacteriaceae*, core members of activated sludge and involved in major ecosystem functions (e.g., organic matter and nutrient removals and floc formation) of activated sludge processes. Further disposal of stabilized sludge in agriculture fields, as fertilizer, might also affect the microbiota of soil if relevant residues of this bactericide remain in the sludge matrix.

The above evidences justify the development of effective analytical methodologies allowing the fast and accurate determination of this biocide. Some specific features of chlorhexidine, such as its strong interaction with negatively charged compounds, large molecular size, and trend to ionize as a mixture of single and double charge species, merit to develop a tailored analytical approach. In general, typical multiresidue methods for water analysis including chlorhexidine are based simply on a SPE using a reversed-phase material such as OASIS HLB and the determination carried out by LC–MS [22, 23]. To the best of our knowledge, no optimized extraction conditions for chlorhexidine analysis in sludge have been reported to date.

The aim of this work was to evaluate for the first time the parameters affecting the yield of matrix solid-phase dispersion (MSPD), as sample preparation technique, for chlorhexidine extraction from freeze-dried sludge samples. Moreover, the effect of the LC column in the performance of the chlorhexidine chromatographic peak was also investigated. The optimized method was applied to determine the residues of this biocide in a selection of samples obtained from different urban STPs, in the Northwest of Spain in different years. The acute toxicity of the pollutant was assessed using in vitro assays considering three different model microorganisms: *Candida albicans*, *Escherichia coli*, and *Staphylococcus aureus*. Also, the potential phytotoxicity was investigated using a standardized germination test using *Sorghum saccharatum*, *Lepidium sativum*, and *Sinapis alba* seeds. Finally, environmental concentrations were compared to in vitro toxicity thresholds.

## Material and methods

### Material, solvents, and standards

Polypropylene syringes (12 mL volume) and polyethylene frits for MSPD extraction (20 µm, 15 mL) were supplied by International Sorbent Technology (Mid Glamorgan, UK). The dispersant sorbent (C18-bonded silica) was obtained from Agilent Technologies (Santa Clara, CA, USA). The co-sorbent, diatomaceous earth, was purchased from Sigma-Aldrich (Milwaukee, WI, USA). PTFE filters, 0.2 µm pore size, from Phenomenex (Cheshire, SK10 2BN, USA) were used.

Methanol (MeOH), HPLC-grade, ethanol, and formic acid (FA) were obtained from Merck (Darmstadt, Germany). Acetonitrile (ACN) was provided by VWR Chemicals (Radnor, PA). Ultrapure deionized water (18.2 MΩ cm^−1^) was obtained from a Genie U system (Rephile, Shanghai, China).

The standard of chlorhexidine (98% purity) was purchased from Sigma-Aldrich and its deuterated analogue (chlorhexidine-d_8_, employed as surrogate standard, SS) was obtained from Toronto Research Chemicals (North York, ON, Canada). Individual stock solutions of each compound were prepared in MeOH. Further dilutions were made in the same solvent.

The commercial Phytotoxkit, employed to investigate the potential phytotoxicity of chlorhexidine to three different model plants (*Sorghum saccharatum* (Sorgho), *Lepidium sativum* (garden cress) and *Sinapis alba* (mustard)), was purchased from Microbiotests (Gent, Belgium).

### Samples and sample preparation

Grab sludge samples were obtained from 23 municipal STPs located in Galicia (Northwest of Spain) in the period 2018–2021. Figure S1 shows the geographic location of the STPs. After reception, samples were frozen, lyophilized, and then stored in glass vessels, at 4 °C, until analysis. MSPD sample preparation conditions were adapted from a previous protocol developed for the screening of emerging pollutants in sewage sludge [24]. In brief, a fraction of 0.5 g of lyophilized sludge, spiked with the SS, was dispersed using 2 g of C_18_ in a glass mortar, for 5 min. The mixture was loaded into a polypropylene syringe containing a polyethylene frit and 1 g of diatomaceous earth. The cartridge was compacted with a second frit on the top. Chlorhexidine was extracted passing MeOH-FA (99:1) (10 mL) through the MSPD cartridge. The obtained extract was passed through a PTFE filter, 0.2 µm pore size, and injected in the UPLC-MS/MS system without any additional treatment.

In order to evaluate the efficiency of the MSPD extraction, spiked samples were prepared by addition of the chlorhexidine standard to a pool of freeze-dried sludge samples (addition level 1 µg g^−1^). Every 5 samples, procedural blanks (without any sludge in the MSPD packed syringe) were prepared in order to evaluate contamination problems during the extraction protocol.

Preliminary experiments were carried out using pressurized liquid extraction (PLE) with MeOH and MeOH-FA (1%) as extraction solvents. As in the case of MSPD, 0.5 g of sludge was dispersed with 2 g of C18 and 1 g of diatomaceous earth added in the bottom of the cell. Extraction was carried out at 90 °C and 1500 psi, during 2 cycles with 100% flush volume. A DIONEX (Sunnyvale, CA, USA) ASE 200 accelerated solvent extractor was used for PLE.

### Toxicity tests

#### Microorganism and culture conditions

Quality control strains of *Escherichia coli* ATCC 25,922, *Staphylococcus aureus* ATCC25923, and *Candida albicans* ATCC 14,053 were used in the acute toxicity assay. The two bacterial strains were routinely cultured on Mueller–Hinton agar (MHA) and broth (MHB) media (Cultimed, Spain) at 37 °C, for 24 h. The fungal strain was cultured on yeast extract-peptone-dextrose agar (YPDA) and broth (YPDB) (Difco) media at 30 °C for 24 h.

The survival of *E. coli*, *S. aureus*, and *C. albicans* following chlorhexidine treatment was evaluated by a colorimetric assay based on the reduction of a tetrazolium salt (MTT method). Briefly, a stock solution of chlorhexidine of 578.5 mg L^−1^ in 96% ethanol was used to prepare a series of two fold solutions at concentrations ranging from 57.9 to 0.06 mg L^−1^ in yeast extract-peptone-dextrose (YPD) and Mueller–Hinton broth (MHC) media. Subsequently, the prepared solutions were dispensed in a volume of 90 µL into the appropriate wells of 96-well U-Bottom microplates. *E. coli*, *S. aureus*, and *C. albicans* suspensions, containing approximately 1 to 2 × 10^8^ colony-forming units (CFU/mL) (OD_620_ of 0.08–0.13), were inoculated to each well in a 1:10 proportion. Colony counts on inoculum suspension were verified by the plate dilution method using MHA and YPDA plates and counting the bacterial/fungal colonies produced. Media with and without inoculum was used as positive and negative control of growth, respectively. MHB and YPDB with chlorhexidine were used as blank. The plates were incubated for 24 h at 37 °C (*E. coli* and *S. aureus* strains) and 30 °C (*C. albicans* strain). Viable bacteria or fungi present in the wells were quantified 30 min after addition of 10 μL of [3(4,5-dimethylthiazol-2-yl)-2,5-diphenyltetrazolium bromide] (MTT, 5 g L^−1^), which is reduced in proportion to the number of viable cells present. Optical density was determined by eluting the dye with dimethyl sulfoxide and the spectrophotometric absorbance measured at 620 nm (Microplate Reader Model 680, BioRad). Experiments were carried out in triplicate. The results were expressed as percentage of mortality using the formula: Normalized mortality (%) = (1 − Absorbance treated samples/Absorbance control) × 100.

#### Phytotoxicity studies

The potential phytotoxicity of chlorhexidine was assessed using two types of dicotyl plants (garden cress (*Lepidium sativum*) and *mustard* (*Sinapis alba*)) and a monocotyl Sorgho (*Sorghum saccharatum*) as models, considering the effects of the biocide in seed germination and in the length of roots after a 3-day germination period. Assays were carried out using two different fractions of a model OECD soil. One of the fractions was used as control, whilst the other was spiked with a solution of chlorhexidine in MeOH at a concentration of 10 µg g^−1^. Both fractions were stored at room temperature for 1 week to permit the evaporation of the organic solvent. Levels of chlorhexidine in the spiked soil were verified by MSPD extraction followed by LC–MS/MS determination. Thereafter, both soil fractions were saturated with water. Germination experiments were carried out in triplicate (three plates were prepared using unspiked and spiked soil samples), placing 10 seeds in each of the plates. Plates were placed vertically and maintained in the dark, at 25 °C, for 3 days before observing the germination of seeds.

#### Ecotoxicological risk assessment

Predicted environmental concentrations in amended soil (PEC_soil_), estimated 1 year after one sludge-dose application, were calculated applying the following equation from the European Commission Technical Guidance Document on Risk Assessment EUR 20,418 EN/2 [25]:

$${\text{PEC}}_{{{\text{soil}}}} = {\text{C}}_{{{\text{sludge}}}} \times {{{\text{APPL}}_{{{\text{sludge}}}} } \mathord{\left/ {\vphantom {{{\text{APPL}}_{{{\text{sludge}}}} } {\left( {{\text{DEPTH}}_{{{\text{soil}}}} \times {\text{RHO}}_{{{\text{soil}}}} } \right)}}} \right. \kern-\nulldelimiterspace} {\left( {{\text{DEPTH}}_{{{\text{soil}}}} \times {\text{RHO}}_{{{\text{soil}}}} } \right)}}$$

where C_sludge_ is the concentration measured in digested sludge expressed as µg kg^−1^ dry mass; APPL_sludge_ is the application rate of dry sludge onto soils (0.5 kg m^−2^ year^−1^ for agricultural soils); DEPTH_soil_ is the mixing depth (0.20 m for agricultural soils); and RHO_soil_ is the bulk density of wet soil (1700 kg m^−3^ for agricultural soils).

### Instrumental analysis

In the current research, a UPLC-QqQ-MS instrument (Waters, Acquity UPLC Xevo TQD) furnished with a Z-ESI source was employed. The capillary voltage was set at 3.10 kV. The source temperature was maintained at 150 °C. Drying gas (N_2_) flow and temperature were 650 L h^−1^ and 200 °C, respectively. Columns tested for chlorhexidine analysis were a Zorbax Eclipse Plus C_18_ rapid resolution (50 mm × 2.1 mm, 1.8 µm) column, acquired from Agilent Technologies (Wilmington, DE, USA), and a Kinetex PS C_18_ (50 mm × 2.1 mm, 2.6 µm) from Phenomenex (Torrance, CA, USA). The columns were protected with a C_18_ 2.1 mm i.d. Security Guard™ cartridge, supplied from Phenomenex. Column and precolumn were maintained at 40 °C. Ultrapure water (A) and MeOH (B), both containing 0.1% FA, were used as mobile phases at a flow of 0.4 mL min^−1^. Under optimized conditions, the Kinetex PS column was used with the following gradient: 5% B (0 min), 20% B (3 min), 100% B (6–7 min), 5% B (7.1–10 min). The injection volume for solvent-based standards and sludge extracts was 0.5 µL. The MS/MS transitions used to determine chlorhexidine and its deuterated analogue are given in Table 1.

**Table 1:** LC-ESI ( +)-MS/MS conditions for chlorhexidine and chlorhexidine-d_8_, linearity, and instrumental LOQ

Compound	Retention time (min)	Precursor ion [M + 2H]^2+^	Cone voltage (V)	Q1 (CE, eV)		Q2 (CE, eV)	Q2/Q1 ratio		Other ions	Calibration curve (5–250 µg L^−1^; *n* = 7 levels)	*R*^2^	LOQ (µg L^−1^)
Chlorhexidine	4.9	253.1	40		170.0 (17)	125.1 (22)	0.45	153.0, 142.1		Slope: 0.0234 ± 0.0006 Intercept: − 0.0577 ± 0.0621	0.997	2
Chlorhexidine-d_8_	4.9	257.1	40		174.1 (17)	125.1 (22)	0.45	157.0 142.1				

A UPLC-QTOF-MS system (Agilent 6550 model, furnished with an electrospray ionization source (ESI), and combined with a 1290 UPLC system from the same supplier) was used to assess the molecular formula of chlorhexidine fragment ions. The instrument was operated in the 2 GHz, extended dynamic resolution mode, with continuous recalibration of the *m/z* axis using the following ions: 121.0508 and 922.0098 (ESI +). Typical mass resolution was 17,000 at *m/z* 322.

## Results and discussion

### Precursor ion and column selection

Ionization of chlorhexidine at ESI sources renders a mixture of [M + H]^+^ and [M + 2H]^2+^ ions, which limits the sensitivity of its LC–MS determination. Furthermore, variations in the relative formation of mono- and di-protonated ions might affect method accuracy. Experiments were carried out using different ESI needle voltages (1.5–3.5 kV) and different compositions of the mobile phase in order to optimize the signal of the pseudomolecular ion. Figure S2 shows the MS spectra corresponding to a standard of chlorhexidine using two different capillary voltages. Whatever the considered ESI ( +) conditions, the [M + 2H]^2+^ ion (*m/z* 253) showed a higher intensity than the [M + H]^+^ one (*m/z* 505); therefore, it was selected as precursor to optimize MRM conditions. Several fragment ions were obtained from the *m/z* 253 precursor: one at *m/z* 170.0480 corresponding to the formula [C_7_H_9_N_3_Cl]^+^ was a monoprotonated ion derived from the break of the end part of the molecule including the chlorinated benzene ring and three amines, and it was used for quantitation. Another one, with *m/z* 125.1073 corresponding to the formula [C_7_H_13_N_2_]^+^, was common to the deuterated form, and therefore, it should correspond to the central-amine part of the molecule because the deuterium atoms were located at the benzene rings. It was used as qualifier. Other ions present in the MS/MS spectra corresponded to *m/z* 153.0214 with formula [C_7_H_6_N_2_Cl]^+^ obtained by NH_3_^+^ loss from the *m*/*z* 170.0480 and to *m/z* 142.1339 with formula [C_7_H_16_N_3_]^+^. All of them are monoprotonated ions.

**Fig. 1:**
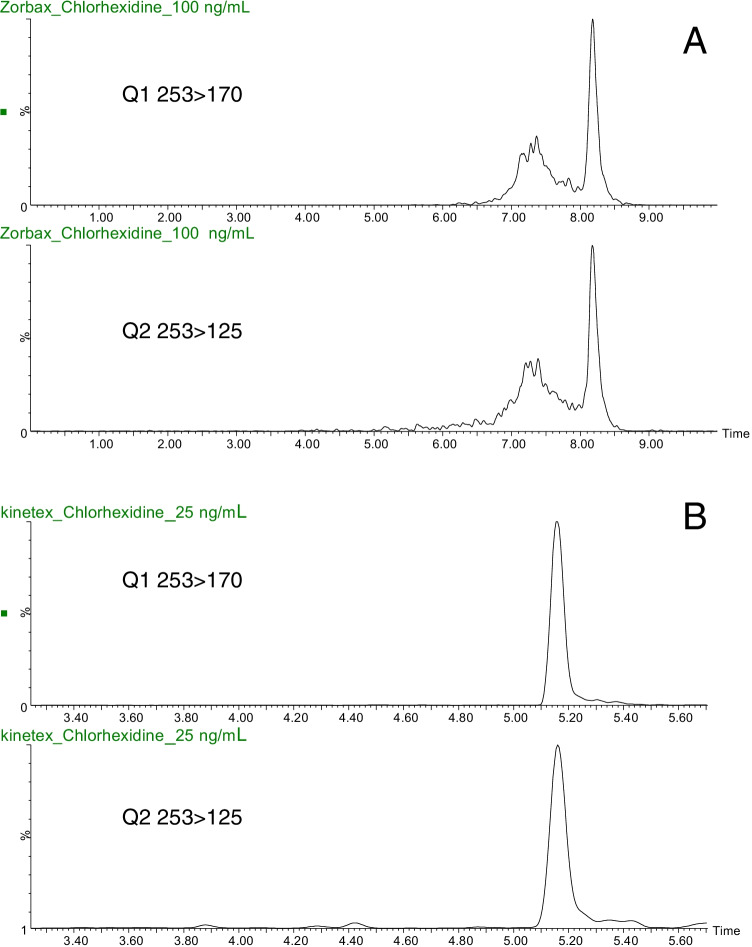
Chromatograms of a standard solution of chlorhexidine in a conventional C18 column (100 µg L^−1^) (A) versus a Kinetex PS one (25 µg L^−1^) (B)

Experimental findings showed that the shape of the chromatographic signal of chlorhexidine in the C_18_-type column degraded very fast, leading to a tailing peak after a few injections. We assumed that the increasing peak tailing was the result of interaction between negatively charged silanol groups in silica particles, and the opposite sign charges in the molecule of chlorhexidine. As alternative, a column specifically designed to deal with strong basic compounds (Kinetex PS column) with a core–shell technology was evaluated to solve the above shortcoming. In this case, the positively charged groups introduced on the surface silica particles of this column avoid the above-described electrostatic interactions. Figure 1 shows the performance of the Kinetex column versus a conventional C_18_ reversed-phase one for a standard of chlorhexidine. Better peak shape and shorter retention were achieved using the Kinetex PS C18 column, which was selected for the rest of the study.

The LOQ and LOD of the QqQ instrument for chlorhexidine were established at 2 µg L^−1^ (Table 1) and 0.8 µg L^−1^ respectively. The LOQ corresponds to the lowest concentration standard providing a signal to noise (*S*/*N*) response of 10 for the less intense MRM transition (Q2), whilst the ratio between responses for qualification (Q2) and quantification (Q1) transitions remains within ± 30% of the average value showed in Table 1. Similarly, for LOD calculation, a signal to noise (*S*/*N*) response of 3 was taken into account. A lineal calibration range between 5 and 250 µg L^−1^ was attained with a determination coefficient of 0.997 using chlorhexidine-d8 at a constant concentration of 50 µg L^−1^ (see Table 1 and Supplementary information Figs. S3 and S4). The inter-day precision for *N* = 10 injections, in three different days, for standard containing100 µg L^−1^ of chlorhexidine was 5%.

The concentrations of chlorhexidine in the extracts from spiked and non-spiked sludge samples were determined using solvent-based standards (5 to 250 µg L^−1^), containing an equivalent concentration (50 µg L^−1^) of deuterated chlorhexidine as SS.

### MSPD extraction and performance of the method

Preliminary experiments with PLE of the sludge reported very low recoveries (25%) when using MeOH as extractant under standard extraction conditions (1500 psi and 90 °C) (Table 2). As a green and simpler alternative to PLE, a MSPD protocol was adapted from a previously reported work dealing with the determination of emerging contaminants in sludge [23]. Following this procedure, the freeze-dried sludge samples (0.5 g) were dispersed with 2 g of C18 over a layer of diatomaceous earth as clean-up sorbent. However, MeOH as eluting solvent failed again to recover quantitatively chlorhexidine from the sludge due to the strong lipophilic-ionic interaction between the compound and the matrix; ACN provided even lower recoveries (9%) (Table 2). Chlorhexidine values of pKa for the basic moieties range from 7.63 (pKa1) to 10.22 (pKa4), and the optimum pH for activated sludge is stated to remain between pH 6.5 and pH 8; therefore, chlorhexidine would be partially protonated in the sludge in the STP and an ionic interaction between the substance and the sludge matrix would take place. Thus, it was necessary to add 1% FA to MeOH to be able to release chlorhexidine from the matrix (Table 2). Probably, formic acid neutralizes some negatively charged sites at the sludge surface enabling the extraction of chlorhexidine. PLE with MeOH-FA provided recoveries of 73% versus 93% provided by MSPD using the same eluant; due to the simplicity of the latter approach, it was selected for the rest of the study.

**Table 2:** Extraction efficiencies for chlorhexidine using PLE and MSPD with different eluents (*N* = 3) and matrix effects for two different injection volumes. Addition level: 2 µg g^−1^

	PLE recovery		MSPD recovery			Matrix effects	
	MeOH	MeOH-FA (1%)	MeOH	ACN	MeOH-FA (1%)	Inj vol. 2 µL	Inj vol. 0.5 µL
Chlorhexidine	25 ± 5%	73 ± 9%	26 ± 4%	9 ± 2%	92.5 ± 7.5%	152 ± 4%	115 ± 3%

**Fig. 2:**
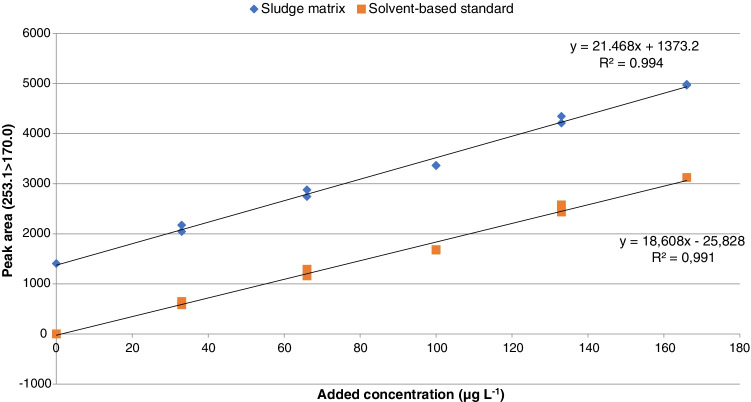
Calibration curves obtained using matrix-matched (blue) and solvent-based (orange) standards. Peak areas for the Q1 transition, without surrogate standard correction, are plotted vs added concentration, duplicate injections

The possible variation in the ionization efficiency of chlorhexidine in sludge extracts versus solvent-based standards was evaluated by comparing the slopes of calibration curves for spiked sludge extracts and solvent-based standards prepared in MeOH-FA (99.9:0.1). An average signal enhancement of 152% was obtained for two tested sludge samples (Table 2). In order to minimize this effect, the injection volume was reduced from 2 to 0.5 µL. As a result, just a slight increase in the response of the sludge matrix (115%) was observed (Fig. 2). The ratio of responses of chlorhexidine and an chlorhexidine-d8 was considered as response variable for the rest of the study.

The overall recoveries for the corrected responses were assessed for three different addition levels over pooled freeze-dried sludge samples: 2000 ng g^−1^, 1000 ng g^−1^, and 500 ng g^−1^. For the lowest addition level, just sludge samples with relatively low levels of chlorhexidine were included in the pooled sludge. Results are compiled in Table 3. They were quantitative in all cases ranging from 94 ± 11% to 120 ± 6% and with a grand mean recovery of 105 ± 6%. The LOQ of the proposed methodology considering 0.5 g of freeze-dried sludge and 10 mL of eluting solvent were established in 40 ng g^−1^ (Fig. S5) and the LOD at 16 ng g^−1^. As further shown, these values were lower than the residues of chlorhexidine found in the processed sludge samples, so verification of estimated procedural LOQ was carried out spiking the deuterated compound in sludge at a concentration of 50 ng g^−1^. Fig. S5 shows the plots of Q1 and Q2 transitions for deuterated chlorhexidine spiked in sludge at 50 ng g^−1^.

**Table 3:** Recoveries of chlorhexidine in three different pooled sludge samples (*N* = 3) with different addition levels

Pooled sludge sample	Recoveries (%)		
	Addition level: 2000 ng g^−1^	Addition level: 1000 ng g^−1^	Addition level: 500 ng g^−1^
1	109 ± 2%	94 ± 11%	101 ± 6%
2	120 ± 6%	101 ± 5%	115 ± 8%
3	97 ± 4%	96 ± 5%	112 ± 8%
Average	109 ± 4%	97 ± 7%	109 ± 7%
Grand mean	105 ± 6%		

### Analysis of real samples

**Fig. 3:**
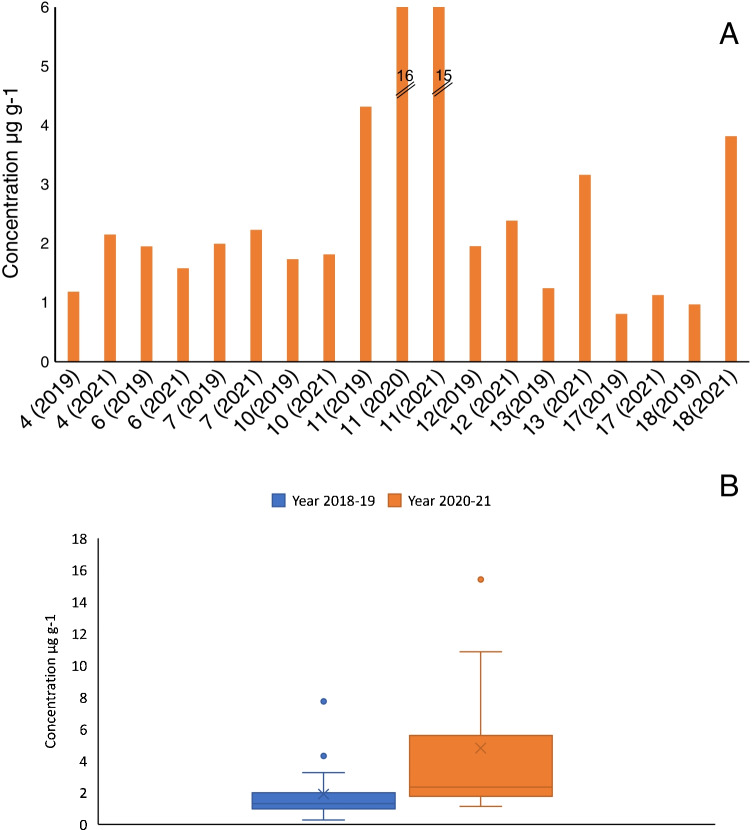
Concentration of chlorhexidine in sludge samples from the same STPs in different years (2019–2021) (A). Box-whisker plots of chlorhexidine residues in sludge in the evaluated period, years 2018–2019 and 2020–2021 (B)

Sludge samples were collected from 23 different STPs in Galicia (Northwest of Spain). Locations of each sampling site are plotted in Fig. S1. Samples were processed with the optimized MSPD protocol and found concentrations were in the range of 0.3–16 µg g^−1^ in the period 2018–2021 (Table S1). These concentrations are in agreement with those found by Östman et al. [16] in Swedish sludges (2.8–19 µg g^−1^). Figure 3A compares the concentrations of chlorhexidine in sludge samples obtained from nine different STPs in, at least, two different years. Overall, concentrations found in the second sampling campaign were similar to, or higher than, those obtained in the sludge from the same STP in different years. Figure 3B shows the box-whisker plots with the distribution of chlorhexidine concentrations measured in sludge samples obtained before and after the outbreak of the COVID-19 disease. As appreciated, higher median and average concentrations were found in samples obtained during years 2020 and 2021, which point out to an increase in the consumption of the antiseptic.

### Ecotoxicity

**Fig. 4:**
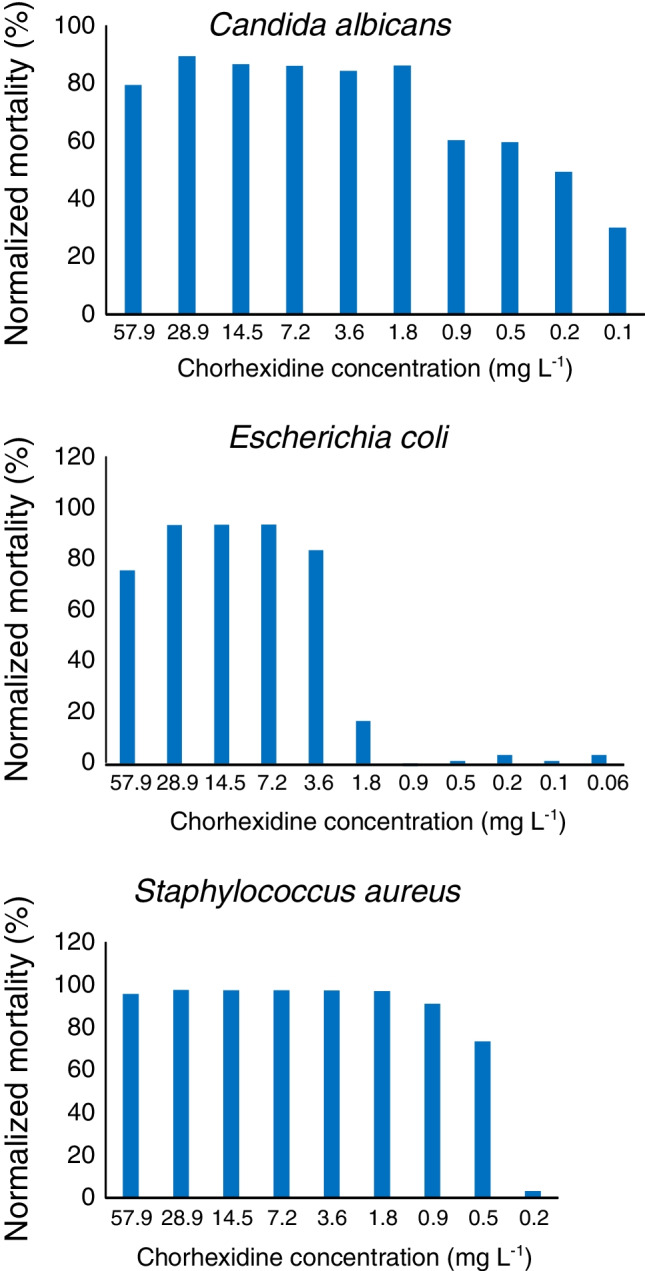
Normalized mortalities for *Candida albicans*, *Escherichia coli*, and *Staphylococcus aureus* versus chlorhexidine concentration

Three microorganisms were tested being exposed to decreasing concentrations of chlorhexidine: *Candida albicans*, *Escherichia coli*, and *Staphylococcus aureus*. The most sensitive of the tested microorganisms to chlorhexidine with lethal threshold concentrations lower than 0.1 mg L^−1^ was *C. albicans* (Fig. 4). *E. coli*, however, remained unaffected until concentrations around 1.8 mg L^−1^ and *Staphylococcus aureus* until 0.5 mg L^−1^ (Fig. 4). Considering a density of in vitro liquid toxicity assay solutions of 1 g mL^−1^, above thresholds are equivalent to 0.1 µg g^−1^, 1.8 µg g^−1^, and 0.5 µg g^−1^ for *C.* a*lbicans*, *E. coli*, and *S. aureus*, respectively. Chlorhexidine was ubiquitous in the sludge samples analyzed and the lowest observed residue, referred to freeze-dried sludge and expressed in µg g^−1^ of dried sludge, was 3 times higher than the acute toxicity threshold measured for *C. albicans*. Furthermore, average concentrations referred to freeze-dried sludge stayed 5 times above the acute toxicity level observed for *S. aureus*, and they are two orders of magnitude higher than PNEC for freshwater sediment reported in the NORMAN data base (6.94 ng g^−1^ dw) [26]*.* Calculated PEC_soil_ (0.44–23.5 ng g^−1^) are also at a similar level as the PNEC in freshwater sediment. In summary, chlorhexidine residues existing in STPs sludge might be high enough to modulate the microbiological communities existing either in STPs or in sludge amended soils.

Regarding plants toxicity, a test was carried out with *Sorghum saccharatum*, *Lepidium sativum*, and *Sinapis alba* in reference OECD soil spiked with chlorhexidine. In this case, chlorhexidine did not present toxicity towards these plant species even with a concentration as high as 10 µg g^−1^ (Fig. S6). Thus, considering PEC_soil_, residues estimated from chlorhexidine measured in the processed sludge samples will not affect the germination of vegetables.

## Conclusions

Chlorhexidine has been confirmed to be ubiquitous in sludge samples. Sample preparation comprises MSPD extraction using methanol modified with formic acid in order to completely recover chlorhexidine from the matrix. Chromatographic performance is clearly improved by the use of columns specifically designed to cope with basic compounds. The chlorhexidine precursor duality, [M + H]^+^ and [M + 2H]^2+^, favors the use of the latter ion in terms of intensity. Under optimized conditions, accurate concentration values can be obtained using solvent-based calibration standards. Concentrations up to 16 µg g^−1^ have been registered, and taken into account the experimental acute toxicity to some microorganism, as *C. albicans*, it can be concluded that chlorhexidine could alter the microbial population of the STP sludge and that existing in soil amended sludge. On the other hand, this pollutant would not affect seed germination.

## Supplementary Information

Below is the link to the electronic supplementary material.

Supplementary file1 (DOCX 1.84 MB)
