# Supplementary material for: Chlorhexidine residues in sludge from municipal wastewater treatment plants: analytical determination and toxicity evaluation
Source: Anal Bioanal Chem. 2022 Jul 13;414(22):6571–80. doi: 10.1007/s00216-022-04214-0 (PMC9278323; doi:10.1007/s00216-022-04214-0)
Supplement: Supplementary file 1 — Supplementary file1 (DOCX 1.84 MB) [file 216_2022_4214_MOESM1_ESM.docx]

Supplementary information to manuscript:

**Chlorhexidine residues in sludge from municipal wastewater treatment plants: analytical determination and toxicity evaluation**

M. Cobo Golpe^1^; G. Castro^1^; M. Ramil*^1^; R. Cela^1^; Y. Santos^2^; I. Rodríguez^1^.

*^1^Department of Analytical Chemistry, Nutrition and Food Sciences, IAQBUS - Institute of Research on Chemical and Biological Analysis, Universidade de Santiago de Compostela, R/Constantino Candeira SN, 15782 Santiago de Compostela, Spain*

e-mail:maria.ramil@usc.es

*^2^Department of Microbiology and Parasitology,* *IAQBUS - Institute of Research on Chemical and Biological Analysis, Universidade de Santiago de Compostela, R/Constantino Candeira SN, 15782 Santiago de Compostela, Spain*

**FIGURES:**

Figure S1. Sampling locations in Galicia (Northwest of Spain).

Atlantic Ocean


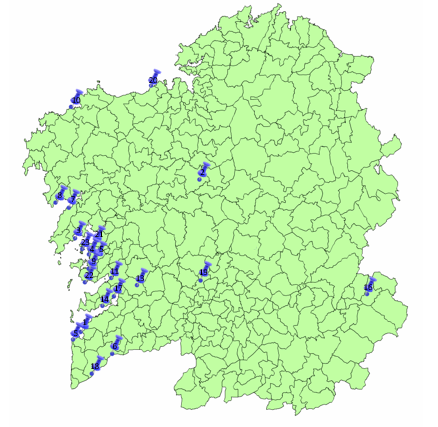


Galicia

Portugal

Spain

Figure S2: Mass spectra for a standard of chlorhexidine using two different voltages of the ESI needle.

|  |
| --- |
|  |

Figure S3: LC-MS/MS chromatogram of a standard solution of chlorhexidine close to the LOQ of the molecule (Concentration: 5 µg L^-1^)

Figure S4: Calibration curve for standard solutions of chlorhexidine (Range: 5-250 µg L^-1^).

Figure S5: Chromatogram corresponding to the extract of a sludge spiked at 50 ng g^-1^ level with deuterated chlorhexidine (chlorhexidine-d_8_).

|  |
| --- |

Figure S6. Lepidium sativum (A) and Sinapis alba (B) exposed to chlorhexidine amended soil (Control: left; addition of 10 µg g^-1^ of chlorhexidine: right).

| **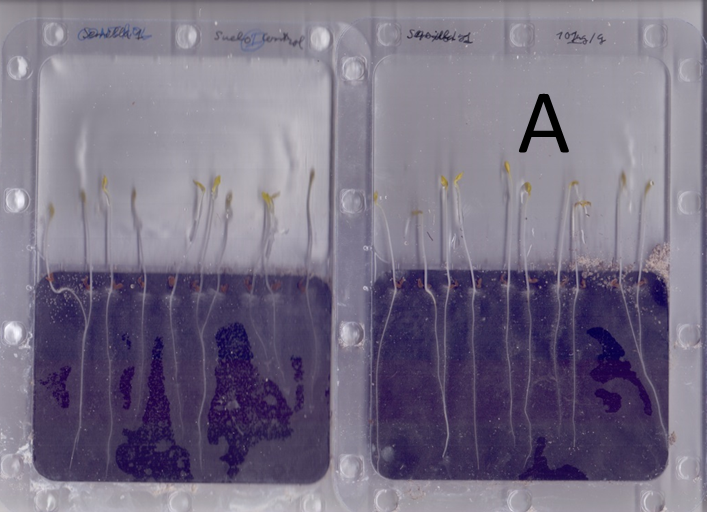** |
| --- |
| **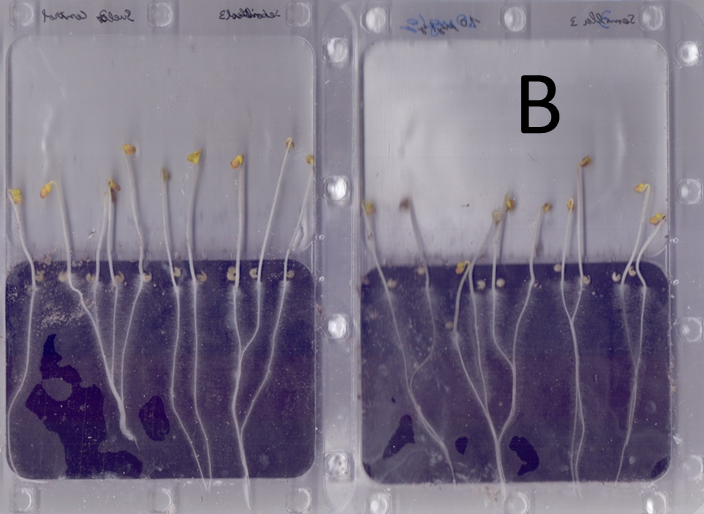** |

**TABLES:**

Table S1: Concentrations of chlorhexidine in different STPs from Galicia (Spain) in the period 2018-2021.

| STP Code | Concentration (µg g^-1^) | | | |
| --- | --- | --- | --- | --- |
|  | Year 2018 | Year 2019 | Year 2020 | Year 2021 |
| 1 | 1.68 |  |  |  |
| 2 |  |  |  | 1.17 |
| 3 | 1.05 |  |  |  |
| 4 |  | 1.19 |  | 2.15 |
| 5 | 0.28 |  |  |  |
| 6 |  | 1.95 |  | 1.58 |
| 7 |  | 2.00 |  | 2.23 |
| 8 | 1.30 |  |  |  |
| 9 | 0.38 |  |  |  |
| 10 |  | 1.73 |  | 1.81 |
| 11 |  | 4.32 | 15.51 | 15.40 |
| 12 |  | 1.96 |  | 2.39 |
| 13 |  | 1.24 |  | 3.16 |
| 14 | 0.79 |  |  |  |
| 15 | 2.31 |  |  |  |
| 16 | 7.74 |  |  |  |
| 17 |  | 0.81 |  | 1.13 |
| 18 |  | 0.97 |  | 3.82 |
| 19 | 3.26 |  |  |  |
| 20 | 10.86 |  |  |  |
| 21 | 3.84 |  |  |  |
| 22 | 2.31 |  |  |  |
| 23 | 1.19 |  |  |  |
